# Embrace Disorder as a Function: Bio-Informed Biomaterials Design Advanced by Intrinsically Disordered Proteins

**DOI:** 10.3390/biomimetics11090680

**Published:** 2026-09-21

**Authors:** Candan Tamerler, Malcolm L. Snead

**Affiliations:** 1 Institute for Bioengineering Research, University of Kansas, Lawrence, KS 66045, USA; mlsnead@ostrow.usc.edu; 2 Department of Mechanical Engineering, University of Kansas, Lawrence, KS 66045, USA; 3 Bioengineering Program, University of Kansas, Lawrence, KS 66045, USA; 4 The University of Kansas Cancer Center, Kansas City, KS 66210, USA; 5 Center for Craniofacial Molecular Biology, Herman Ostrow School of Dentistry of USC, The University of Southern California, Los Angeles, CA 90033, USA

**Keywords:** intrinsically disordered proteins, bio-informed biomaterials, short linear motifs, biomineralization, functional peptide design, machine learning, artificial intelligence

## Abstract

The “lock and key” model of molecular recognition, anchored by the canonical sequence–structure–function paradigm, has successfully served as a basis for designing biomimetic biomaterials for decades. This classical concept associates the function of molecules with a stable three-dimensional structure that recognizes a specific complementary surface. However, biological systems routinely achieve specificity, adaptability, and multifunctionality through protein domains that never adopt a single stable fold. Intrinsically Disordered Proteins (IDPs) and Intrinsically Disordered Regions (IDRs) represent an underutilized functional repertoire in which conformational plasticity, short linear motifs, and phase-separation capacity underpin behaviors that rigid-fold proteins cannot replicate. This Perspective Article extends the molecular biomimetic design framework to the broader range of IDPs/IDRs roles—as effectors triggering downstream signaling, as motion-directing elements interacting with external partners, and as molecular assemblers that organize dynamic complexes while largely avoiding the steric constraints that structured proteins impose. Sequence plasticity, which is greater in disordered domains than in structured proteins, further expands this versatility. Metamorphic and moonlighting proteins further extend the sequence-to-function landscape by encoding multiple folds or functions within a single chain. We discuss how this bio-informed framework guides peptide-based biomaterials design by exploiting the dynamic, multivalent interactions of IDPs. Artificial Intelligence (AI)-guided approaches and machine learning strategies—from supervised classifiers to reinforcement learning loops—that are integrated with experimental feedback to navigate within these high-dimensional design landscapes of intrinsically disordered bio-informed materials systems are provided. Next-generation bio-informed material design must meet challenges driven by clinical, environmental, and industrial needs. Expanding protein matrix in bio-informed material design may enable mimicking complex, dynamic and hierarchical biological functions and materials that are found in Nature. Embracing disorder may even expand functions beyond addressing current challenges. Moving beyond the single-structure paradigm that has dominated the biomimetic field, embracing disorder in biological protein repertoire may give rise to emerging functions in bio-informed biomaterials. These functions are truly adaptive, modular, resourceful, responsive, and sustainable.

## 1. Introduction

Biological materials achieve a combination of properties—hierarchical organization, adaptive mechanical response, and self-repair—that synthetic materials have yet to fully replicate [1,2,3]. At the molecular level, these properties emerge largely from proteins that serve as the foundational structural and functional molecules of biomineralization, adhesion, signaling, antimicrobial defense, self-assembly, regulatory switch, evolutionary adaptation, and many other multivalent interactions [4,5,6,7]. For much of the twentieth century, the conceptual understanding of protein function rested on a complementary pillar: the sequence dictates structure, and structure dictate function. This concept was introduced by Christian Anfinsen’s work on ribonuclease A in the 1950s, for which he won the Nobel Prize in 1972 [8]. The thermodynamic hypothesis for the native 3D structure of a protein has been described by the global free energy minimum. The well-defined 3D structure has been considered as the physical basis for function through the structure-function coupling, implying one-sequence leads to one-fold and one-function. This paradigm has proven enormously productive, enabling the rational engineering of biomolecular-based materials design, guiding how we adapt the role of protein when mimicking Nature. Current understanding, however, suggests the existence of substantial disorder in eukaryotic proteomes with the discovery of Intrinsically Disordered Proteins (IDPs), and Intrinsically Disordered Regions (IDRs) frameworks from the ensemble paradigm concept [9,10,11,12,13,14]. Now, integrating IDPs and IDRs into the design of functional materials to recapitulate biological inspiration is the next frontier in molecular biomimetics.

For decades, the operational paradigm of structural biology hypothesized that biological function is inseparably from a unique 3D structure encoded by a specific amino acid sequence. This conceptual bias, i.e., one gene-one protein-one function, carried an implicit assumption—that disorder is a deficiency rather than a notable feature that could be operationalized. Mounting evidence from proteomics, structural biology, and cell biology challenges this assumption profoundly [15,16,17,18,19,20,21,22]. In fact, with the current knowledge, intrinsic disorder is considered as a highly evolved feature empowering polyvalency to create a switch-like ultrasensitive biological responsiveness. Intrinsic disorder is an indication of multifunctionality enabling an access to differently folded functional domains [23]. Nature uses intrinsic disorder across life forms as a highly conserved evolutionary strategy with a positive correlation to increased organismal complexity [22,24,25]. Estimates suggest that 30–40% of eukaryotic proteins contain extended disordered regions—with some analyses reporting up to 50% depending on the prediction algorithm and threshold—with a substantial fraction of these entirely devoid of a stable fold under physiological conditions [22,26,27].

Far from being non-functional, these IDPs and IDRs mediate critical processes including signal transduction, transcriptional regulation, liquid–liquid phase separation, and the formation of membraneless organelles or biological condensates [28,29,30,31]. Their intrinsic disorder imparts multifaceted roles and diverse functions: from being effectors, to acting as entropic chains, assemblers of large complex assemblies, directing motion, as well as being involved with interactions with external partners [32]. Their binding is often driven by polyelectrostatic interactions generated by their large dynamic ensemble of IDPs that results in highly cooperative and non-linear ultrasensitive binding. Their polyvalent interactions respond to even a small change in the environment, shifting their non- or weak binding to maximum binding. This is very different than rigid folded proteins, which rely on stereochemical pairing based on one primary binding site available per functional domain. IDPs play a fundamental role in mitigating and managing steric hindrance in biological systems through their flexible nature, allowing them to act as linkers, connectors, modulators, and decouplers. This behavior is unlike the proteins with rigid folding, which results in potential blockage of access to binding sites. The flexible tethering property of IDRs enables the domains to rotate and adapt to specific topography for the counterpart without sterically crowding each other. The rapid fluctuations of the dynamic conformational ensembles result in their larger hydrodynamic volume compared to folded rigid proteins and serve as an exclusion zone for non-specific interactions by other proteins. Molecular shielding provided by the entropic bristles behavior of IDPs keeps the protein in a functional, soluble state while preventing aggregation as they navigate within molecularly, highly crowded areas [33]. Their fuzzy complexes holding the highest structural disorder allow them to mold their conformation to fit their partners by extending flexible chains and to overcome steric barriers through a motion similar to fly casting [34,35]. These proteins and IDRs also act as molecular assemblers as they coordinate multiple binding partners to form a higher-order protein complex and promote large dynamic molecular complexes [11].

**Figure 1:**
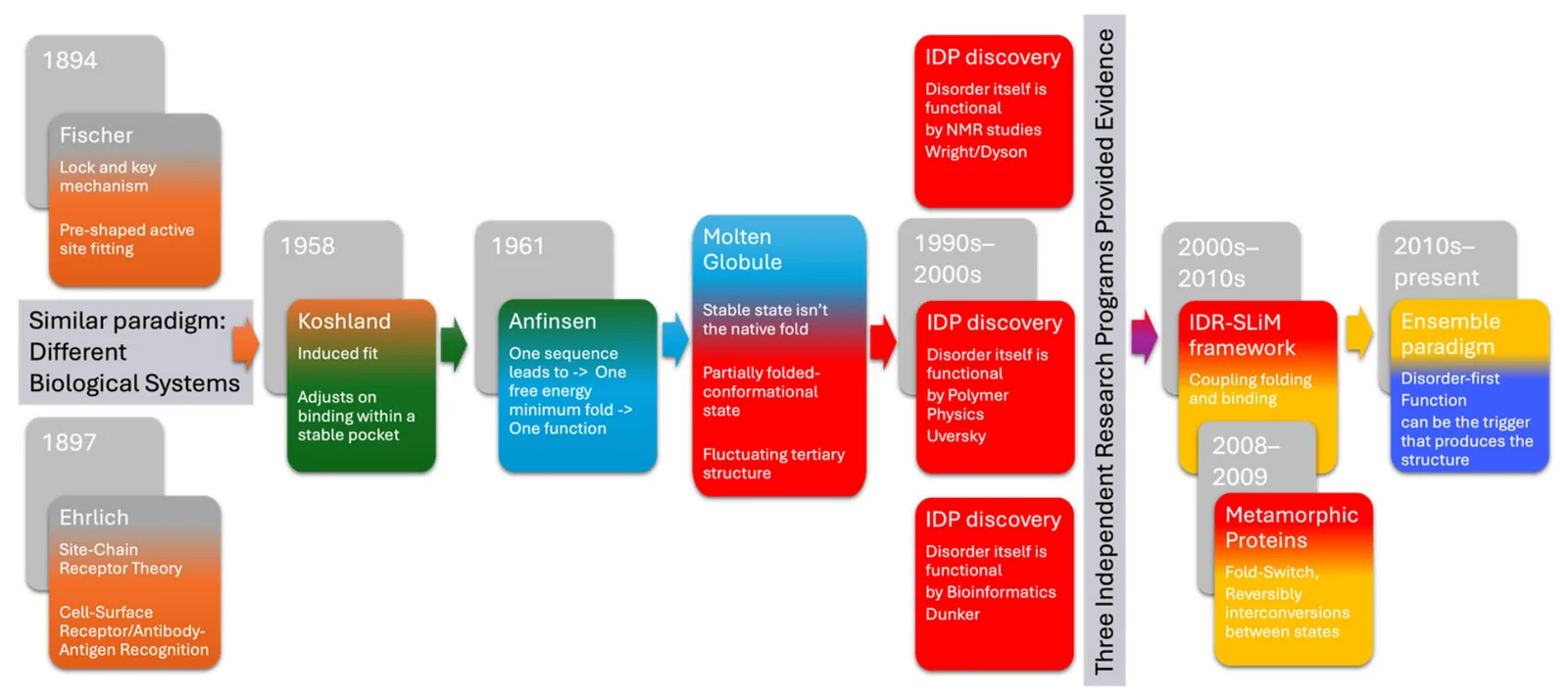
Timeline of conceptual shift in understanding protein functions.

For decades, this operational paradigm of structural biology served to explore enzyme active sites, allosteric pockets, and binding grooves. It built upon the highly ordered structural state for function. The root of this assumption goes back to the structural complementarity, i.e., the lock-and-key model of enzyme–substrate recognition, introduced by Emil Fischer in 1894 [36]. Fischer’s “lock-and-key” metaphor, introduced in 1894, captured an essential truth (Figure 1): molecular recognition requires geometric and chemical complementarity between interacting partners [4]. In this view, enzyme and substrate surfaces possess complementary shapes stabilized by non-covalent interactions—electrostatic ion–ion forces, dipole interactions, hydrophobic contacts, and van der Waals forces. While providing a conceptual picture for researchers, the model had a major influence on interrelating concepts from chemistry, biology, and medicine [37,38]. The concept of specificity in the form of rigid geometrically complementary binding sites was introduced by Paul Ehrlich in 1897 as a model of immunity, referring to cell-surface receptor/antibody-antigen recognition [36,39]. This foundational model accounts for the exquisite specificity of enzyme–substrate pairs and provided the conceptual basis for competitive inhibition pharmacology and structure-based drug design. The models of Fischer and Ehrlich form the founding pillars of rigid complementarity. Yet the lock-and-key model fails to accommodate a body of observations accumulated steadily since the mid-twentieth century. Major deficiencies of the model include the assumption of rigidity, failing to recognize the dynamic nature of molecules and the complex binding behavior with multiple substrates.

Koshland’s induced-fit hypothesis (1958) provided the first major revision, positing that substrate binding triggers conformational changes in the enzyme that optimize geometric complementarity [40]. More radical revisions followed with the recognition that many proteins exist as conformational ensembles even in the absence of ligands, and that binding may involve conformational selection from a pre-existing ensemble followed by induced fit [41]. Altogether, these concepts support that the one sequence holds one native structure with slightly adjustable rigidity, which was formalized thermodynamically by Anfinsen’s work providing principles governing protein folding [8]. The concept of partially folded compact intermediates was introduced by “Molten Globule” indicating a presence of cooperatively folded non-native states, a fluidic uncooperative side-chain packing with the retained compactness [42,43]. The Monod–Wyman–Changeux (MWC) model proposed the existence of the cooperative ligand binding with two conformational states, i.e., low affinity and high affinity, in oligomeric proteins [44]. The Koshland–Nemethy–Filmer (KNF) model was proposed as a sequential allostery extending the original induced fit model from single-subunit to multi-sub-unit oligomers [45]. Both MWC and KNF models were not correct but reflected an ongoing debate. The evidence for an intermediate in protein folding provided by Ptitsyn’s group built upon the availability of rigorous high-resolution Nuclear Magnetic Resonance (NMR) spectra of cytochrome c reported by Wada’s group. Wada named the “molten globule”, to curate the partially folded state a stable functional intermediate step, a concept that challenged the view that “structure precedes function”.

Van der Waals forces, with their steep distance dependence, emerge as particularly pervasive contributors to shape refinement across diverse recognition systems [46]. With the induced-fit model, more sophisticated simulation approaches were developed to explore molecular recognition at a larger energy landscape. Molecular docking has gained a key role in structure-based drug design, serving to identify and predict the affinity and orientation of targeted interactions for the proteins in the search. Docking strategies evolved from rigid-body docking, to flexible docking, to match induced fit, and the application was dependent on the processing time needed to analyze the complexity of these interactions, with increasing computational power leading to advancement. To sample the vast conformation space, search algorithms ranging from stochastic to systematic or simulation-based methods were utilized. Identifying the best binding modes utilizes a variety of scoring functions, including force-field-based, empirical, or knowledge-based, which were applied to estimate the binding free energy and account for the fitness of the interactions. The evolution from lock-and-key model to induced-fit model then expanded to one native fold, a framework which enabled investigators to advance understanding and emulate the dynamic nature of biological systems.

At the turn of the 21st century, a subsequent convergence of methodologies by research teams led to the concept “disorder is a function” (Figure 1). Pioneering NMR work on disordered regions folding upon binding by Wright and Dyson led to the “coupled folding and binding” concept [47]. The sequence-based disordered predictors by Dunker and colleagues shifted the understanding of natively unfolded regions to a new level and forced these regions not to be considered as artifacts [48]. Uversky introduced physicochemical understanding to IDPs providing their distinct region of sequence space using charge-hydropathy plots [49]. Since 2000, other research projects expanded the IDPs and IDRs content exemplified by germline P granules in *C. elegans* being demonstrated to behave as liquid droplets [50], and the observation of phase transitions by multivalent signaling proteins [51]. Wright and Dyson’s body of work extending over two decades continued to provide a methodological foundation for IDPs through NMR spectroscopy [52]. IDPs could not be revealed by crystallography, which diffracts the ordered conformation. This was seen as a technical problem for years. NMR spectroscopy enabled providing a spectrum over the entire conformational ensemble over a weighting average across the populated structures, and revealed disorder. The core tool, PONDR (Predictor of Natural Disordered Regions), built by Dunker and colleagues, enabled scaling a genome wide argument by applying disorder to large protein sets and made a statistical case on disorder not being randomly scattered across the proteome. Uversky’s core tool on the charge-hydropathy plot and foundational biophysical characterization provided a first principles physiochemical explanation, and moved the field from empirical observation to predictable consequences of amino acid composition [20].

Establishing “disorder exist” elevated the research to search for operational toolkits towards predictable, searchable sequence features [10,53]. The short linear motifs (SLiMs), 3–11 amino acid residues were shown to be embedded in IDRs to mediate selective interactions, while SLiMs binds with a low affinity, drives a fast on/off binding, and plays a role as a fast switch rather than a permanent lock [54,55,56,57]. Another interesting group featured molecular recognition features, roughly 5–70 residues, embedded in longer disordered regions with a role of binding to a partner as they undergo through disorder to order transition [53,58,59,60,61]. With the introduction of fuzzy complexes, IDRs were also shown to have dynamic interactions even in the bound state. These functional complexes are stable in composition but remain fuzzy as the functionally relevant state, not a transient intermediate [12,62,63,64,65].

Embracing the IDPs and IDRs potential into the design of biomaterials unlocks innovative approaches and potentially overcomes many of the challenges in designing bio-hybrid materials and delivery systems. Flexible and dynamic interfaces with respect to binding sites, addressing kinetic barriers, and environmental parameters, including pH, ionic strength and temperature, and modulating the dynamic interactions triggered within the systems can be inspired by the range of IDPs and IDRs interactions. These could be extended to understand and address other stress induced interactions observed in biological systems in relation to materials [66,67]. The practical implication of IDRs-inspired biomaterials design is significant: if molecular recognition is inherently dynamic and context-dependent, then engineered materials that rely solely on complementarity may fail when deployed in the chemically variable, mechanically dynamic environments of living tissues and other dynamic systems. This realization motivates the search for design principles that are themselves flexible and adaptive.

Changing of the reciprocal site during binding is a conceptual bridge to fully embrace disorder as a functional property. Once one accepts that proteins are flexible and undergo conformational changes upon binding, the question becomes quantitative: how much disorder is compatible with function, and under what conditions does conformational freedom confer a selective advantage? The answers indicate that a wide spectrum of disorder—from localized flexible loops in otherwise rigid proteins to entirely unfolded polypeptide chains—can be functionally productive, provided that the sequence encodes appropriate interaction hot spots and regulatory logic.

This perspective is organized around three interlocking themes. First, we present a protein matrix on the protein functionality that progresses from traditional proteins with rigid folds at the base to fully disordered IDPs at the apex, encompassing metamorphic “transformer” and moonlighting proteins as intermediate categories. Second, we provide a snapshot of the IDRs-derived motifs in diverse tissues that attracted interest in biomimetic design. Third, we evaluate the computational and experimental strategies, particularly AI-guided discovery and machine learning (ML) approaches, being developed to navigate the complex functional materials and interface design landscapes that disordered biomolecular systems present.

## 2. Unifying Framework for Functional Protein Repertoire

**Figure 2:**
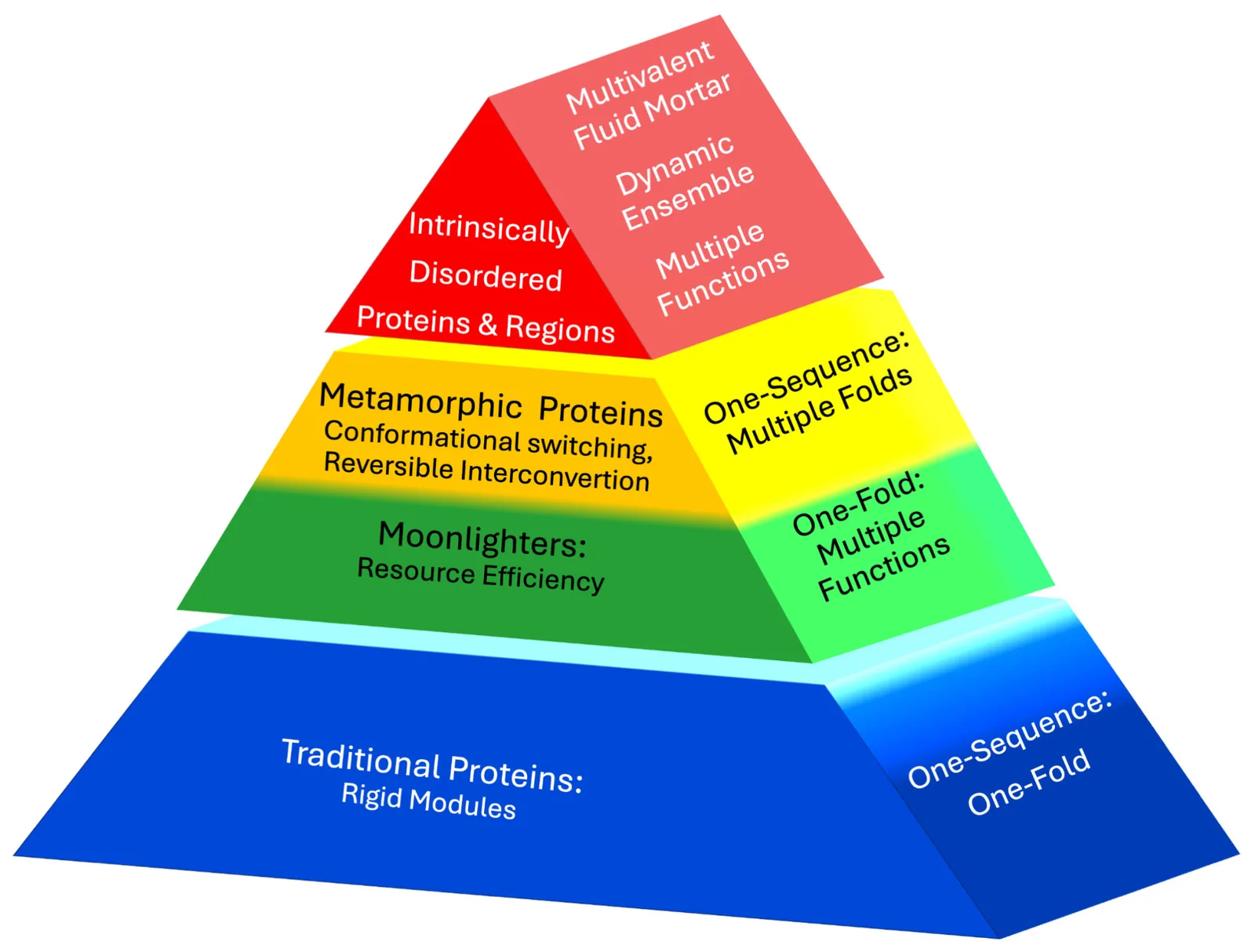
Protein Matrix to mimic biology’s engineering meta-solution for functional engineering.

To organize the continuum from rigidity to disorder in a manner useful for biomaterials design, we propose a unifying framework shown as a pyramid of protein functionality comprising three tiers (Figure 2). This framework is not intended as a strict classification system or an alternative nomenclature, but rather as a conceptual framework for reasoning about which design strategies are appropriate for which functional demands. The pyramid selection is expected to reflect the knowledge accumulated with respect to each tier in order to emphasize the ongoing discovery of the functional repertoire for the IDPs and IDRs.

At the base of the hierarchy are classically folded proteins characterized by stable, cooperative tertiary structures and high sequence conservation. These proteins embody the one sequence–one-fold–one function paradigm and achieve their functions through precisely positioned active-site residues or binding surfaces [11,18]. The cooperative folding of these proteins means that function is tightly coupled to fold integrity—a property that confers both exceptional performance under optimal conditions and vulnerability to environmental perturbation. Consequences to perturbation sensitivity can propagate and collapse folding due to destabilization of one region, expose hydrophobic regions leading to often irreversible unfolding, or cause steric hindrances in specific side-chain geometry. Elevated temperatures, mechanical stress, or chemical denaturation can destroy functional folding, and produce a non-recoverable aggregated state by eliminating the folding, which has a direct relevance to biomaterials, medical device and delivery systems, biofabrication, manufacturing, and sterilization processes [68,69]. Despite the limitations, rigid proteins remain indispensable in biomaterials contexts where highly optimized, specific activity is required, and environmental conditions are well-controlled. Enzymes incorporated into diagnostic biosensors, antibody-functionalized surfaces, and growth factors incorporated into bone graft substitutes or theranostic delivery systems exemplify productive applications of this tier [70,71,72].

The intermediate tier of the hierarchy accommodates a group of proteins that expand the sequence-to-function landscape beyond the one sequence–one-fold–one function constraint. In this tier, we focus on moonlighting and metamorphic proteins.

Moonlighting proteins maintain a single predominant fold but are competent to perform multiple distinct biological functions, often in different subcellular compartments, cell types, or physiological contexts [73]. One of the best-characterized examples of protein moonlighting is the glycolytic enzyme phosphoglucose isomerase (PGI). PGI functions extracellularly as a neurotrophic factor and cytokine while being a cytosolic glycolytic enzyme. Extracellularly, this enzyme can function as an inflammatory mediator or modulator depending on the biological processes, and takes different names, i.e., neuroleukin factor in neurotrophic context or autocrine motility factor in tumor biology or a maturation factor in differentiation context. The extracellular neurotrophic factor was originally discovered as ‘neuroleukin’ [74] before cloning established its identity with the glycolytic enzyme [73,75]. The fascinating mechanistic view about protein moonlighting, as in this example, is that the enzymatic activity is not required for the extracellular functions. The same molecules can drive cell migration or act as a pentose phosphate pathway connector. Another example is the crystallin protein component of the lens of animal eyes [76], while aldehyde dehydrogenase and transketolase are the putative corneal crystallins in mammals, and gelsolin is used as the corneal crystallin in zebrafish [77]. Yet a moonlighting protein is the splicing isoform of mammalian amelogenin (AMELX), the most abundant protein contributing to the formation of the enamel bioceramic covering teeth, which can also act as an activator of the canonical Wnt/beta-catenin signaling pathway leading to bone formation [78]. Moonlighting confers remarkable resource efficiency in evolution—a single gene product contributes to multiple pathways. From a design perspective, moonlighting behavior suggests that carefully engineered sequence features can encode orthogonal functions within a single molecular scaffold. When translating this information into bio-informed biomaterial design, functional consequences are massive; mechanistically unrelated functions can be incorporated into engineering design, and functions can be unloaded depending on the environmental triggers.

Metamorphic proteins, a recently discovered class of biomolecules, have intrinsic plasticity latent in their own energy landscape inherent to the amino acid sequence [79]. Metamorphic proteins encode a single amino acid sequence capable of adopting two or more distinct stable folds in response to environmental triggers such as changes in pH, redox state, ligand concentration, or oligomeric context [61,79,80]. Mechanistically, meta-morphic sequences require a folding funnel to permit interconversions where the conformers can move easily from one basin to another triggered by these environmental perturbations [61]. The plasticity here is not imposed by an external chaperone or a covalent modification, it is inherent to the primary sequence. Earlier studies focused on the chemokine lymphotactin (XCL1), a well-characterized example: it interconverts between a canonical chemokine fold and an alternative dimeric structure with distinct receptor-binding properties, with the equilibrium regulated by ionic strength and temperature [81]. The same sequence leads to two topologically unrelated folding outcomes simply driven by environmental conditions. Similarly, the protein KaiB, identified in cyanobacteria, flips its folding synchronized with the Earth’s rotation, as a part of the biological clock for circadian rhythm [82]. Yet, the critical turning point for metamorphic proteins was the identification of metamorphic candidates resulting in an estimation of ~4% of known proteins [83]. Follow-on computational analysis combined with domain-based searches led to an estimation of 5% of PDB-deposited structures as metamorphic [84]. Furthermore, the NusG/RfaH family work extended the understanding of metamorphism to a family-wide trait [85]. The implications of these findings range from a deep-learning revolution to application-driven predictions and design. Importantly, metamorphic proteins retain function in multiple structural states—switching mediates a change in function rather than a loss of function. While metamorphic proteins and their functions will improve the protein based targeted therapeutics and delivery systems; they will also advance molecular machines and self-assembled systems through bio-enabled and bio-informed molecular switch designs. For biomaterials design, metamorphic proteins and their derived peptides offer an underexplored design logic: materials that respond to the chemical cues present in wound environments—local acidification, chemokine signals, elevated reactive oxygen species, or protease activity—by switching between states with different therapeutic, inflammatory/pro-resolution, antimicrobial, or mineralizing properties. Stimulus-responsive multifunctionality is intrinsically encoded in the sequence rather than achieved through complex chemical conjugation. This intrinsic plasticity may make design tractable in translating a metamorphic mechanism into biomedical materials applications.

At the apex of the hierarchy, IDPs and IDRs lack a single stable fold under physiological conditions, existing instead as dynamic conformational ensembles. Stated in a fanciful way, IDPs and IDRs act as multivalent fluid mortars by integrating signals, facilitating phase-separations, and enabling “plug-and-play” ability through their versatility. Their functional properties are encoded not in a fixed three-dimensional structure but in the statistical properties of this ensemble: the time-averaged exposure of interaction surfaces, the probability of adopting transiently structured states upon binding (‘coupled folding and binding’), and the capacity to undergo liquid–liquid phase separation into membraneless organelles, often called biomolecular condensates [7,86]. IDRs possess characteristic sequence compositions—enriched in charged residues, proline, serine, and glycine, and depleted in bulky hydrophobic residues that drive hydrophobic core formation—that distinguish them from folded protein sequences [7,87]. Within this low-complexity context, functional specificity is achieved through SLiMs: sequence stretches typically 3–15 amino acids in length that serve as compact, combinatorially recompilable interaction modules [56,88]. SLiMs recognize binding partners, post-translational modification sites, or localization signals with affinities and specificities appropriate for transient, regulatable interactions.

The architecture of IDRs can be further parsed into ‘stickers’ and ‘spacers’ [89]. Stickers are interaction hot spots—typically SLiMs or hydrophobic patches—that mediate intra- and intermolecular contacts. Spacers are intervening sequences that modulate overall chain dimensions, flexibility, and the accessibility of stickers. The arrangement and identity of stickers and spacers determine the phase behavior, binding valency, and mechanical properties of the disordered chain. This modular architecture is directly amenable to rational engineering of functional bio-informed materials.

A unifying conceptual perspective is that disorder functions as a ‘meta-adaptive’ solution: it enables biological systems to respond robustly and flexibly to a wide range of environmental demands without requiring foreknowledge of the specific challenge. This is precisely the property most needed in materials designed for deployment in complex, variable, living environments.

## 3. Merging IDPs/IDRs Inspired Blueprint into Bio-Informed Materials Design

The modular architecture is directly amenable to rational engineering of functional bio-informed materials by offering functional advantages. First, IDRs can bind multiple structurally distinct partners through the same or overlapping sequence segments, enabling hub-like connectivity in signaling networks [89,90]. Second, the entropic cost of folding upon binding can be used to tune interaction affinity and specificity independently [28,34]. Compared to structure proteins, there is no cooperative fold to break, minimum hydrophobic collapse driving force to disrupt. Instead, the conformation ensemble respond to perturbation shifts as a distribution. Third, IDRs are less susceptible to irreversible denaturation than are rigid proteins because they lack a cooperative fold to unfold; they can recover native ensemble properties upon return to permissive conditions [7,91]. Functional robustness across a wider range of pH, ionic strength, and temperature is particularly relevant for biomaterials applications where materials may be exposed to manufacturing stresses or the harsh environments of the mammalian body ranging from the challenging conditions found in a tumor environment, to the unique demands of the oral cavity [92].

**Figure 3:**
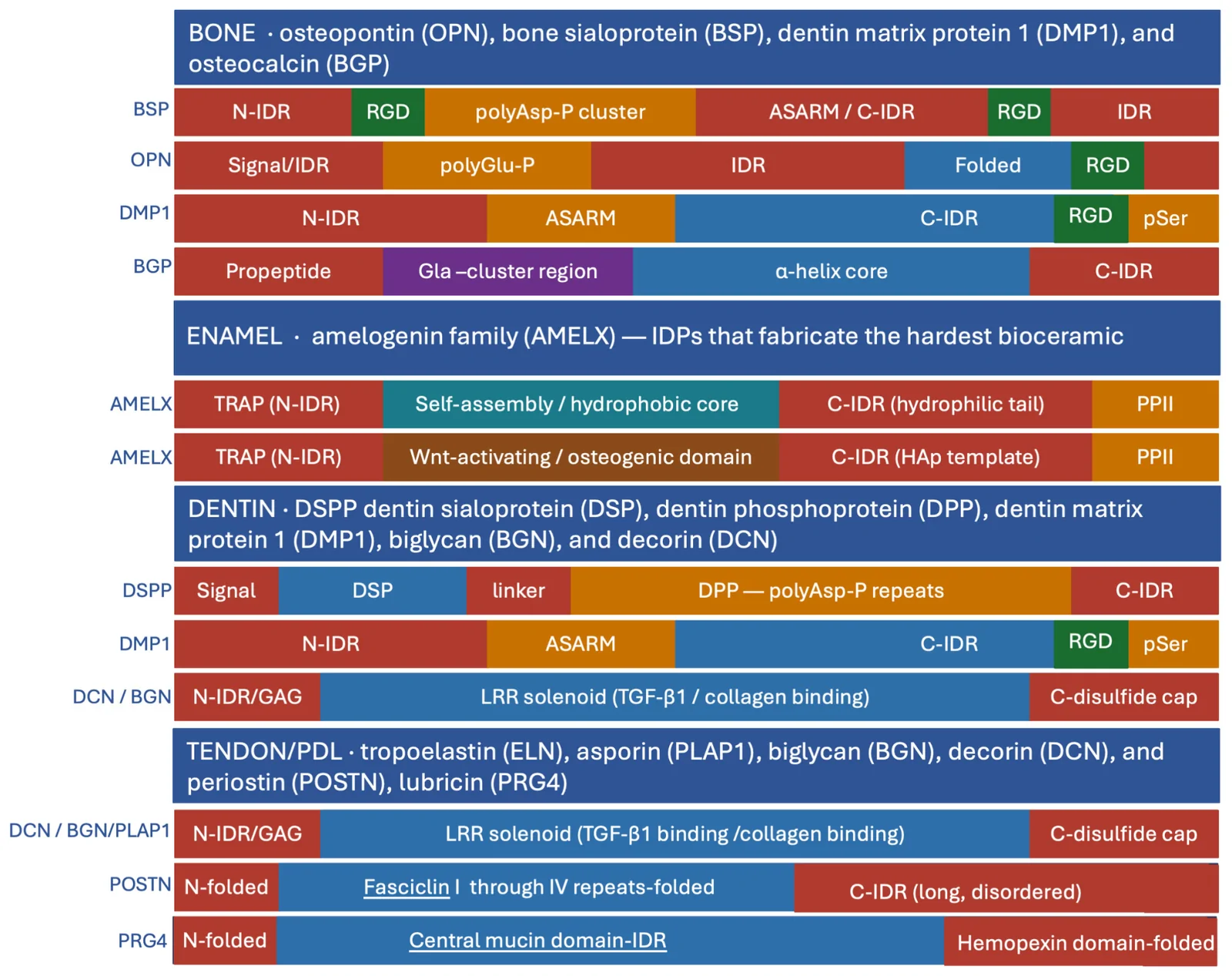
Snapshot of Intrinsically Disordered Domains as Functional Engines of Biomineralization, Fibrillogenesis, and Cytokine Sequestration Modulating Inflammation.

Figure 3 provides a snapshot of the IDRs-derived motifs from proteins playing a critical role in diverse tissues that attracted interest in biomimetic design. We present examples of full length IDPs and IDRs as inspirational biological functional engines that are utilized in biomineralization, fibrillogenesis, and inflammation control across bone, enamel, dentin and tendon.

These IDPs and IDRs-derived domains include nucleating or growth regulating clusters (DPP: dentin phosphophoryn, OPN: osteopontin, BSP: bone sialoprotein, BGP: osteocalcin) used in dentine and bone biomineralization; RGD cell-adhesion sequences (OPN, BSP, DMP-1: dentin matrix protein 1), which regulate tissue development, cell adhesion, wound repair, and inflammatory tissue remodeling [93,94,95,96], and the enamel amelogenin splicing isoform leucine-rich amelogenin peptide (LRAP C-terminal) operating as a hydroxyapatite (HAp)-templating protein that moonlights as a Wnt-activator, and the ASARM motif found in several SIBLING (small integrin-binding ligand N-linked glycoprotein family) including OPN and DMP-1 [78,97,98]. Some of these domains have been condensed into 14–22mer solid-binding peptides that recapitulate the three functional targets: hydroxyapatite (HAp) surface recognition, collagen fibrillogenesis, and cytokine sequestration [99,100]. In the case of tendon and the periodontal ligament (PDL), tropoelastin (ELN), asporin (PLAP1), biglycan (BGN), decorin (DCN) and lubricin (PRG4) contain IDR regions that serve to keep these connective tissues compliant and non-mineralizing.

The SLiMs-based modularity of IDRs provides an inspiration for a direct blueprint in designing peptide modularity in engineering bio-informed materials. By assembling SLiMs with distinct functional properties—mineral nucleation, collagen binding, antimicrobial activity, cell adhesion—into a single chimeric sequence connected by flexible spacers, it is possible to engineer peptides with multiple encoded functions [101,102,103]. This approach mirrors the evolutionary logic by which IDRs themselves evolved through recombination of modular elements, and it offers a design space that is both vast and systematically explorable.

Critical design parameters needed for deployment in materials design include the spacing between functional motifs, the conformational tendencies of spacer sequences, and the net charge and amphipathicity of the assembled construct. Computational tools for predicting these properties have advanced, but experimental validation remains essential, particularly considering the heterogeneous complexity of biological interfaces dictated by the application [104,105].

Liquid–liquid phase separation (LLPS) driven by IDPs has emerged as a fundamental mechanism for organizing cellular biochemistry into membraneless condensates with distinct compositional and rheological properties [11,106]. From a biomaterial’s perspective, LLPS represents an underexplored mechanism for self-organizing material architectures. IDPs-based coacervates can sequester ions, concentrate enzymes, and create microenvironments with distinct pH and ionic conditions—properties with direct relevance to biomineralization, where local supersaturation and pH control are critical for crystal nucleation and growth [106,107,108,109].

Bioinspired coacervate systems, drawing on the design logic of IDPs involved in biomineralization such as amelogenin and the intrinsically disordered enamel matrix proteins, offer a promising avenue for engineering controlled mineral deposition at the interfaces of dental and bone bioceramic tissues. Amelogenin, the primary protein of the secretory enamel matrix, contains both a hydrophobic core capable of self-assembly and disordered C-terminal and N-terminal domains that mediate self-assembly and mineral-surface interactions [109,110,111,112,113,114,115,116]. Amelogenin-derived peptide sequences have demonstrated remineralization activity in vitro and represent a clinically translatable embodiment of bio-informed IDPs design [117]. Enamel is the hardest biological tissue, organized as a hierarchical array of carbonated hydroxyapatite crystallites—enamel rods—whose formation is orchestrated by a transient protein matrix dominated by amelogenin [110,118,119]. Amelogenin self-assembles into nanospheres and nanoribbons through interactions mediated by its hydrophobic core and is processed by matrix metalloproteinase 20 (MMP20) and kallikrein-4 (KLK4) during amelogenesis [109]. Its C-terminal domain, disordered in solution, mediates interactions with the apatite mineral surface. Following enamel maturation, amelogenin is fully degraded, leaving a protein-free, highly mineralized tissue incapable of intrinsic regeneration. Amelogenin-derived peptides incorporating the leucine-rich amelogenin peptide (LRAP) and shorter functional fragments have demonstrated the capacity to nucleate oriented hydroxyapatite growth on demineralized enamel surfaces in vitro and in animal models [97,111,120]. These constructs function through SLiMs-like mineral-binding sequences that modulate crystal nucleation and growth kinetics—a direct translation of IDPs design logic into a clinical application targeting repair of caries lesions [111].

The oral cavity presents exceptional challenges for biomaterials: cyclic mechanical loading, wide pH excursions (from ~3.5 during acid challenge to ~7.4 at baseline), enzymatic degradation by salivary and bacterial proteases, and a diverse, dynamic microbial community [121]. Disordered peptide constructs that retain activity across a wide pH range, resist proteolytic degradation through sequence engineering, and modulate their conformation in response to pH changes are ideally suited to this environment. Here is where the critical expansion could be guided by AI-enabled research discovery and innovation. ML-guided peptide design has enabled the optimization of enthalpic interaction profiles under acidic pH conditions, yielding candidates with enhanced binding to mineral surfaces and collagen substrates under the conditions prevailing at the dentin–adhesive interface [111,122,123,124,125,126].

The biomaterials field has begun to engage with this reality, moving from biomimetic strategies toward bio-inspired and now bio-informed design—the systematic translation of biological molecular principles into engineered systems [127,128]. Nowhere is this transition more consequential than in the design of biomaterials for craniofacial and dental applications, where tissues such as enamel, dentin, alveolar bone, and the periodontal ligament present multiscale, heterogeneous bioceramic architectures whose formation is orchestrated by disordered or conditionally structured proteins including amelogenin, ameloblastin, and dentin sialoprotein [98,110,118,119,129]. Next-generation biomaterials need to satisfy a patient-centered process to be incorporated into material design and bridging disease state to materials performance in response to the specific biochemical environment of that disease. The current understanding indicates the involvement of IDPs and IDRs in governing the interactions in these challenging environments. While this perspective presents examples selected to address the materials and delivery systems needs for craniofacial and dental applications, the manuscript’s contribution to biomimetics lies in its explicit translation of biological molecular design logic—particularly the principles of structural disorder, modularity, and phase separation—into actionable frameworks for engineering next-generation bio-informed materials.

## 4. AI-Enabled Discovery and ML Strategies for Intrinsically Disordered Functions on Peptide Informed Biomaterials Design

The identification of function for IDPs and IDRs has been tremendously accelerated in recent years with the convergence of AI and ML [130]. They have transformed the IDRs and IDPs studies extensively by bridging the gap between sequence data and dynamic physical behavior. ML models have continued to serve as the engine in decoding disorder patterns in diverse sequence domains, and interpretation of ensemble data while AI has continued to provide the physics-grounded generative framework to relate the patterns to functions.

Early predictors scored each residue based on its physicochemical propensity scales, e.g., charge-hydropathy (CH-plot) and FoldIndex to relate the level of disorder [131,132]. Here hydrophobicity, net charge, and aromatic content, or any prior information on the biological function, were used for propensity classification. Second-generation propensity classification, i.e., PONDR (Predictor of Natural Disordered Regions) variants, IUPred (Intrinsically Unstructured Protein Prediction), ESpritz (Efficient Sequence-based prediction system), DisEMBL (Disorder by European Molecular Biology Laboratory), and GlobPlot (Globularity Plot), included sliding window statistical ML classifiers [133,134]. Data training used experimentally characterized IDPs including NMR and X-ray data with missing residue labels. Convolution neural networks (CNNs) and Transformers have been used to extract the rules from sequences [130,135]. The CAID (Critical Assessment of Protein Intrinsic Disorder Prediction) benchmarking has contributed tremendously to the tool expansion and served to standardize the field [136]. Protein language models, e.g., ESM-2 (Evolutionary Scale Modelling-2), serve to create large embeddings to capture statistical likelihood of disorder, and are trained on a large number of sequences [137,138,139]. This is a continuously growing area, with disorder prediction methods being released [140]. Another concurrently evolving capability was introduced by the feature embedding layer linking sequence to a learned representation. Meta-morphic proteins certainly resulted in a blind spot in the deep learning revolution. Despite the success of predicting folded proteins using AI/ML methods including AlphaFold2, prediction of highly dynamic conformational ensemble of IDRs and IDPs has been challenging [141]. AlphaFold 3 and AlphaFold-Multimer provided improved predictions to model disorder–order transition. AlphaFold-Multimer was recently shown to identify the IDRs residues as interaction-prone and to predict their binding modes with their specific binding partner [142,143].

Several sets of tools have been developed and used for classification and generative categories. While classification provides embedding function label, generative layer links sequence to the ensemble. With the expanding databases and the prediction tools, the field also moved very rapidly beyond “disorder or not” to predict biological function within the disordered domains. Molecular recognition features, short linear motifs, disordered protein binding regions, and fuzzy complexes using deep learning and protein language models attracted attention in the extraction pipeline steps [144].

Training data typically reflects crystalline, folded proteins for which high-resolution structural data are most easily obtained. Compositional discovery in ordered crystalline materials has benefited greatly from large, high-quality datasets amenable to supervised learning [145,146]. Disordered systems, by contrast, present sparse, heterogeneous datasets with conformational complexity and multiscale coupling that conventional feature representations capture poorly. The challenges are far beyond the limited data size; they extend to labeling, i.e., crystal structure would miss the disordered regions, and evaluation metrics would be scarce and non-uniform due to ensemble dynamics. In addition to the generalizability issue due to limited data, AI/ML models are further challenged by limited interpretability and transferability. While AI/ML continues to revolutionize the way we study function relationships in disordered proteins and expand on using the experimental and simulation data, there is certainly a need to expand the experimental approaches to understand IDRs and IDPs protein conformations, dynamics, conformations, and interactions with their binding partners. The experimental techniques could be achievable in different environments mimicking cellular environment, within a context of biomolecular condensates, or under osmotic stress conditions [144]. There has been several biophysical and functional characterization to interrogate the IDRs and IDPs, including HDX-MS (hydrogen deuterium exchange mass spectroscopy), CD (circular dichroism), NMR, FRET (fluorescence resonance energy transfer), SAXS (small-angle X-ray scattering), Cryo-EM (cryogenic electron microscopy), and other direct or indirect biochemical assays where function can be detected [85,147,148]. Experimental data will immensely help to expand the limited training data sets for disordered proteins and the regions. Training data typically reflects crystalline, folded proteins for which high-resolution structural data are most easily obtained. Compositional discovery in ordered crystalline materials has benefited greatly from large, high-quality datasets amenable to supervised learning [145,146]. Disordered systems, by contrast, present sparse, heterogeneous datasets with conformational complexity and multiscale coupling that conventional feature representations capture poorly. The challenges are far beyond the limited data size; they extend to labeling, i.e., crystal structure would miss the disordered regions, and evaluation metrics would be scarce and non-uniform due to ensemble dynamics. In addition to the generalizability issue due to limited data, AI/ML models are further challenged by limited interpretability and transferability.

A practical taxonomy of ML approaches applicable to disordered peptide design maps available data quality to algorithmic strategy. When labeled data exists with clear activity classifications, supervised learning—including support vector machines, random forests, and deep neural networks—can identify sequence features associated with desired activities. As data become sparse, semi-supervised and unsupervised approaches, including variational autoencoders and generative adversarial networks, enable exploration of sequence space beyond existing datasets [149]. Reinforcement learning, in which iterative experimental feedback drives model refinement and sequence selection, is particularly well suited to the early-stage design of novel peptide scaffolds where no high-quality labeled dataset exists [150,151,152].

A persistent concern in ML-guided biomaterials design is the interpretability of model predictions. Black-box models may achieve high predictive accuracy while encoding feature relationships that fail to generalize across sequence space. In the context of peptide design that mimics specifically IDPs and/or IDRs functions, features driving the outcome can be quite complex to analyze; iterative approaches with experimental validation can be costly without providing an answer for the functional call; therefore, throughput is limited; non-interpretable predictions can lead to expensive experimental dead ends.

The preference for ‘gray box’ model architectures—including genetic algorithms that encode explicit evolutionary optimization logic and provide interpretable fitness functions—reflects a deliberate trade-off between raw predictive performance and the credibility and actionability of model outputs [55,56]. The trajectory toward ‘white box’ models—fully interpretable architectures that encode known physicochemical relationships combined with multiple additional features—represents the longer-term aspiration of the field. These models can be used as hybrid approaches and offer to bridge the gaps and allow the refinement of the AI/ML-generated data analyzing ensembles with the specific descriptors. These descriptors could be focused on ensemble content for mineralization or binding in response to a trigger rather than solely on the structure of the peptide. This conceptual shift to ensemble function rather than a peptide/protein structure would serve well for bio-informed materials design.

A recognized limitation of current ML frameworks for disordered peptide design is their tendency to encode sequence-level features while neglecting the mesoscale and systems-level context that modulates peptide function in situ. A peptide that performs optimally in a homogeneous aqueous environment may behave very differently when incorporated into a polymer matrix, adsorbed onto a mineral surface, or deployed in the presence of competing biomolecules [153]. These functions can be further challenged depending on the environmental triggers due to a diseased state or a stress because of a change in the environmental conditions.

Future AI/ML frameworks will need to encode context across hierarchical levels—from the molecular sequence features that predict the IDPs and IDRs functions and decode them to discover functional domains and their triggered conditions. Conceptual shift is the sequence–ensemble–function inference for the ML strategy in the domain design. Then, transferring from disorder predictors with the multi-features may lead to downstream property prediction, determining intrinsic peptide properties, through the mesoscale structural features of the material matrix, to the systems-level biological environment that determines ultimate function. The sticker-and-spacer logic that determines peptide behavior at the molecular scale can be extended, by analogy, to the design of mesoscale material architectures.

A pronounced conceptual and linguistic gap separates the communities of biologists and materials scientists working on related problems at biological interfaces. The development of truly bio-informed, rather than merely biomimetic, materials will require sustained engagement across these disciplinary boundaries, co-developing not only new materials but new conceptual vocabularies adequate to their complexity [127]. Interdisciplinary venues bridging biology and materials science serve as critical bridges, providing shared language and collaborative frameworks for exactly this kind of transdisciplinary translation.

## 5. Conclusions

The design of structural biomaterials that mimic Nature has achieved remarkable success. Nacre, glass sponges, spider silk, bone, teeth (ranging from human to radula), gecko feet, lotus leaves, butterfly wings, diatoms, Venus’s flower basket, bamboo, and wood are among the most studied examples of Nature’s structural biomaterials repertoire [1,154,155,156,157,158]. As we continue to learn from Nature, its IDPs and IDRs will be among the next frontiers for exploration and adaptation in the next generation of bio-inspired, biologically informed biomaterials design.

The shift from rigid-fold to disorder-embracing frameworks for protein-based biomaterials design represents more than a refinement of existing paradigms—it constitutes a foundational reconceptualization of how biological function is encoded at the molecular level and how that encoding can be translated into engineered systems. The hierarchical framework presented here—from rigid cooperative folders, through metamorphic and moonlighting intermediates, to fully disordered IDPs—provides a structured vocabulary for identifying which design strategies are appropriate for which functional demands.

Key design principles derived from this framework include the exploitation of SLiMs for combinatorial multifunctionality, the engineering of sticker and spacer architectures for tunable binding valency and phase behavior, and the design of stimulus-responsive conformational switching for materials that adapt to physiological cues.

AI/ML-guided strategies calibrated to available data quality—from supervised classifiers for well-characterized activities, to reinforcement learning for early-stage exploration of novel scaffolds—provide the computational tools necessary to navigate the high-dimensional, sparse design landscapes intrinsic to disordered systems. The evolution toward interpretable, *“white box”* model architectures will be essential for ensuring that ML-guided designs are not only predictively accurate, but mechanistically coherent and experimentally actionable.

Looking forward, with the convergence of biology, materials science, and AI/ML disciplines merged with predictive design around the principle of functional disorder, this adaptation constitutes one of the most promising frontiers in biomaterials—a frontier that extends to other technological materials as well. Realizing this potential will require sustained interdisciplinary collaboration, investment in high-quality experimental datasets, and willingness to revise canonical paradigms in light of evidence that Nature’s most adaptive materials are built not from rigid perfection but from principled disorder.

## Data Availability

No new data were created or analyzed in this study. Data sharing is not applicable to this article.
